# Pentraxin 3 (PTX3) Is Associated with Severe Sepsis and Fatal Disease in Emergency Room Patients with Suspected Infection: A Prospective Cohort Study

**DOI:** 10.1371/journal.pone.0053661

**Published:** 2013-01-14

**Authors:** Raija Uusitalo-Seppälä, Reetta Huttunen, Janne Aittoniemi, Pertti Koskinen, Aila Leino, Tero Vahlberg, Esa M. Rintala

**Affiliations:** 1 Department of Infectious Diseases, Satakunta Central Hospital, Pori, Finland; 2 Department of Internal Medicine, Tampere University Hospital, Tampere, Finland; 3 University of Tampere Medical School, University of Tampere, Finland; 4 Fimlab Laboratories, Tampere, Finland; 5 Department of Clinical Chemistry, Turku University, Turku, Finland; 6 TYKSLAB, Turku Univeristy Hospital, Hospital District of Southwest Finland, Turku, Finland; 7 Department of Biostatistics, Turku University, Turku, Finland; 8 Department of Hospital Hygiene and Infection Control, Turku University Hospital, Turku, Finland; Harvard Medical School, United States of America

## Abstract

**Background:**

Early diagnostic and prognostic stratification of patients with suspected infection is a difficult clinical challenge. We studied plasma pentraxin 3 (PTX3) upon admission to the emergency department in patients with suspected infection.

**Methods:**

The study comprised 537 emergency room patients with suspected infection: 59 with no systemic inflammatory response syndrome (SIRS) and without bacterial infection (group 1), 67 with bacterial infection without SIRS (group 2), 54 with SIRS without bacterial infection (group 3), 308 with sepsis (SIRS and bacterial infection) without organ failure (group 4) and 49 with severe sepsis (group 5). Plasma PTX3 was measured on admission using a commercial solid-phase enzyme-linked immunosorbent assay (ELISA).

**Results:**

The median PTX3 levels in groups 1–5 were 2.6 ng/ml, 4.4 ng/ml, 5.0 ng/ml, 6.1 ng/ml and 16.7 ng/ml, respectively (p<0.001). The median PTX3 concentration was higher in severe sepsis patients compared to others (16.7 vs. 4.9 ng/ml, p<0.001) and in non-survivors (day 28 case fatality) compared to survivors (14.1 vs. 5.1 ng/ml, p<0.001). A high PTX3 level predicted the need for ICU stay (p<0.001) and hypotension (p<0.001). AUC^ROC^ in the prediction of severe sepsis was 0.73 (95% CI 0.66–0.81, p<0.001) and 0.69 in case fatality (95% CI 0.58–0.79, p<0.001). PTX3 at a cut-off level for 14.1 ng/ml (optimal cut-off value for severe sepsis) showed 63% sensitivity and 80% specificity. At a cut-off level 7.7 ng/ml (optimal cut-off value for case fatality) showed 70% sensitivity and 63% specificity in predicting case fatality on day 28.In multivariate models, high PTX3 remained an independent predictor of severe sepsis and case fatality after adjusting for potential confounders.

**Conclusions:**

A high PTX3 level on hospital admission predicts severe sepsis and case fatality in patients with suspected infection.

## Introduction

Severe sepsis is a important disease associated with significant mortality [1]. Early diagnosis and stratification of sepsis patients is difficult but essential, because early interventions and appropriate antimicrobial treatment can be life saving [2], [3]. Biomarkers could play an important role in this process if they can indicate sepsis or its severity [4], [5]. C-reactive protein (CRP), the prototype of short pentraxin, has been widely used, but its specificity as a diagnostic tool is limited [6], [7], and it is also a poor prognostic marker [8]. Procalcitonin (PCT) has been proposed as a more specific etiologic and prognostic marker than CRP [9], [10], although its value has also been challenged [11]. The search for better biomarkers of sepsis thus continues.

Pentraxin 3 (PTX3) is the prototype of the long pentraxin family [12], [13]. It differs from CRP in terms of gene organization and localization, ligand recognition, producing cells and inducing signals [14], [15], [16]. CRP is produced in the liver, whereas PTX3 is an inflammatory mediator produced by various cells in peripheral tissues. PTX3 is an acute-phase protein whose plasma concentrations increases rapidly in various inflammatory conditions, including sepsis [17]. It plays an important role in the early phase of inflammation: it recognizes microbial moieties, activates the classical pathway of complement and facilitates recognition by macrophages and dendritic cells [18]. PTX3 has an important role in regulating the innate immune response by contributing to the opsonization and clearance of apoptotic or necrotic cells [19].

In one previous study a high PTX3 has been shown to predict sepsis and severe disease in febrile patients admitted to emergency department [20]. In critically ill patients PTX3 correlated with severity of disease and infection [21]. High PTX3 has been shown to be associated with mortality in severe sepsis [22] and bacteremic patients [23]. High PTX3 has also been found to be an early indicator of shock in severe meningococcal disease [24].

Aim of the present study was to evaluate the usefulness of plasma PTX3 determination in early stratification and in predicting the development of severe sepsis and mortality in a large and unselected cohort of patients with suspected infection admitted to the emergency room. PTX3 results are compared to CRP and PCT levels. We found that a high PTX3 concentration predicted severe disease and poor outcome in this cohort.

## Methods

### Patients

The aim here was to evaluate early prognostic and etiologic utility of PTX3 in patients with suspected infection in an emergency room setting. The same cohort of patients has previously been involved in three published studies [25], [26], [27]. Patients were recruited in Satakunta Central Hospital, a 350-bed secondary care hospital in Western Finland serving the Satakunta Hospital District with a population of 240 000 inhabitants. It is the only hospital in the area with an emergency department and an intensive care unit. The study was approved by the ethical review board of the Satakunta Hospital District. Written consent was obtained from patients or close relatives.

The study cohort comprised adult patients admitted to the emergency room with suspected infection, from whom a clinician had decided to take samples for blood cultures. Enrolment took place over a 14-month period in 2004 to 2005. To ensure written informed consent and interview within 24–48 hours, only patients admitted between Sunday 7 a.m. and Wednesday 3 p.m. were enrolled. Prior to the study, a pre-evaluation of the target population was conducted to ensure the representativity of the cohort. The assessment covered 1551 consecutive patients from whom blood cultures had been taken in the emergency department. The rate of positive findings was 8.3% and case fatality by day 28 after admission 6.7%. No significant differences were noted between patients in respect of study days and other days or between the study and the pre-evaluated populations regarding age, gender, rate of positive blood cultures or mortality.

Blood samples for the study were taken upon admission concurrently with the blood culture samples. Blood was collected into two 10-ml EDTA tubes (plasma) and two 7-ml serum tubes (serum). The EDTA tubes were kept on ice until centrifugation at room temperature using 2500 G-force 10–15 min. Plasma and serum were transferred in 1–2-ml aliquots to CryoPure® (Sarstedt, Germany) tubes. These were stored at −70°C until assayed.

A structured interview was undertaken by the investigator or research nurse 24–48 hours after admission. Highest body temperature, lowest blood pressure, highest pulse, respiratory rates were recorded daily on days 1–7. Symptoms and clinical signs, Glasgow coma scale, risk factors for sepsis, underlying diseases and diagnosis at admission were recorded, likewise duration of stay in intensive care and in hospital. Potential organ failure on days 0–28 (respiratory, cardiovascular, renal, hematological, hepatic or central nervous system), overall case fatality (day 28) and sepsis-attributable case fatality were recorded. Final diagnoses, source of infection and trauma or other possible reasons for inflammation were taken from medical reports. A follow-up check was made by phone 3 months and one year after enrolment.

Blood samples for the study were taken from 609 patients. Fifty-five patients (or close relatives) refused to participate, and their blood samples were destroyed. Fifteen were excluded from the analysis: one due to a missing blood sample at admission, 11 due to incomplete data for classification as to whether they had bacterial infection or not, and three who had SIRS and organ dysfunction but no bacterial infection (one with epidemic nephropathy and two with myocardial infarction). Two patients were excluded as their plasma samples had run out. The final study material thus comprised of 537 patients.

### Laboratory Methods

The PTX3 concentration in EDTA plasma was determined using a commercial solid-phase enzyme-linked immunosorbent assay (ELISA) (Quantikine® DPTX 30; R&D Systems Inc., Minneapolis, USA). The level of procalcitonin (PCT) in plasma was measured by immunochemiluminometric assay (ECLIA) in a Modular E170 automatic analyzer (Roche Diagnostics GmbH, Mannheim, Germany), and the level of C-reactive protein (CRP) in plasma by an immunoturbidimetric assay using a Modular P800 automatic analyzer (Roche Diagnostics GmbH).

### Statistical Analysis

An SPSS package (version 15) was used for statistical analyses and a two-sided p-value <0.05 was taken as cut-off for statistical significance. Categorical data were analyzed by *X^2^* test or Fisheŕs exact test when appropriate, and nonparametric continuous data by Mann-Whitney U-test or Kruskal-Wallis test. SAS (version 9.2) was used for logistic regression analysis. A logistic regression model, adjusted for potential confounders, was used to study the independent effect of high PTX3 activity on mortality and severe sepsis. Odds ratios (ORs) were expressed with their 95% confidence intervals (CI) when appropriate. The accuracy of a high PTX3 value in predicting severe sepsis and case fatality was assessed using ROC curves [28]. In this method, a test which is perfect has 100% sensitivity and no false positives (1-specificity = 0) and will have an area under the curve (AUC) of 1.0, whereas a test of no diagnostic value would have an AUC of 0.5. The 95% confidence intervals were calculated. The Youden index with the highest sum of sensitivity and specificity was used to select the optimal cut-off for analysis. Correlations between PTX3, CRP and PCT were analyzed using Spearman’s rank analysis.

## Results

Demographic data on the study population are shown in Table 1. Table 2 gives the distribution of the cohort into five study groups on the basis of ACCP/SCCM Consensus Conference definitions [29]. The median PTX3 level in all patients was 5.4 (range 0.3–514 ng/ml).

**Table 1:** Demographics of study population (N = 537).

Characteristics	
Age, median (range)	64 (18–100)
Gender (female/male)	227/310
Obesity (BMI≥30)^a^	119 (30.5%)
Alcohol abuse^b^	25 (4.7%)
Smoking (current smoker)	126 (23.5%)
Diabetes (type 1 and 2)	81 (15.1%)
Malignancy (solid or haematological)	95 (17.7%)
Rheumatic diseases	50 (9.3%)
Chronic renal insufficiency^c^	18 (3.4%)
Cardiovascular disease^d^	289 (53.8%)
COPD or asthma^e^	108 (20.1%)
Operation six months previously	75 (14.0%)
Device^f^	82 (15.3%)
Continuous medication^g^	389 (72.4%)
Continuous cortisone treatment^h^	59 (11.0%)
Blood cultures^i^	
Positive (clinically significant)	47 (8.8%)
Positive (contamination)	4 (0.7%)
Blood cultures taken after antimicrobialtreatment has started	136 (25.4%)

a body mass index. Data available on 390 patients.

b alcoholism was diagnosed or patient had previously been treated for alcohol-induced disease.

c plasma creatinine concentration constantly more than 170 µmol/l (5 patients had chronic dialysis treatment).

d continuous medication for cardiovascular disease (i.e. hypertension, arteriosclerosis or other cardiovascular disease).

e continuous medication for asthma or COPD.

f joint or heart valve prosthesis or pace-maker (does not include dental implants).

g continuous medication for a chronic disease.

h continuous systemic cortisone treatment (daily dose more than 10 mg of oral prednisolone).

i Blood cultures were taken from 536 patients.

**Table 2:** Plasma pentraxin 3 (PTX3) in patients admitted to emergency room with suspected infection stratified by diagnosis groups (N = 537).

Diagnosis group	Criteria	Pentraxin 3 (ng/ml)median (quartiles)
1. No SIRS, no bacterial infection (N = 59)	Patients with no SIRS^a^ (less than two SIRS criteria atadmission +/−24 hours), nor documented^b^ orprobable c bacterial infection	2.6 (1.4–5.8)
2. Bacterial infection, no SIRS (n = 67)	Patients with documented or probable bacterialinfection, but no SIRS (less than two SIRS criteria atadmission +/−24 hours)	4.4 (2.3–9.3)
3. SIRS, no bacterial infection (n = 54)	Patients with SIRS (at least two SIRS criteria atadmission +/−24 hours), but no documented orprobable bacterial infection	5.0 (2.3–13.2)
4. Sepsis (n = 308)	Patients with sepsis (SIRS and documented orprobable bacterial infection but no organdysfunction due to sepsis)	6.1 (2.8–14.2)
5. Severe sepsis (n = 49)	Patients with severe sepsis (sepsis with signsof organ failure, i.e. disturbed perfusion,metabolic acidosis, oliguria or neurological disorders)	16.7 (4.7–54.7)

a SIRS (Systemic Inflammatory Response Syndrome): At least two of the following conditions. 1. Temperature >38°C OR <36°C, 2. Heart rate >90 beats per minute. 3. Respiratory rate >20 breaths per minute or PaCO2<32 mmHg (4.3 kPa). 4. White blood cell count >12×10^9^/l or <4×10^9^/l or >10% immature (band) forms).

b Documented bacterial infection: Microbiologically confirmed bacterial infection (either pathogenic bacterial growth in blood culture or in normally sterile tissue or the same usually less pathogenic bacterium (e.g. Staphylococcus epidermidis) in two different samples).

c Probable bacterial infection: A clinician suspected bacterial infection and either infection focus was confirmed or antimicrobial treatment was started and the response to treatment supported bacterial infection.

Differences between the five groups were studied using Kruskal-Wallis test (p<0.001).

PTX3 values showed a positive correlation with PCT (r = 0.562, p<0.001) and CRP (r = 0.222, p<0.001), WBC (r = 0.236, p<0.001) and creatinine concentration (r = 0.171, p<0.001). A weak negative correlation was documented with platelet count (r = −0.209, p<0.001).

Plasma PTX3 values stratified by demographics, underlying conditions and clinical data are presented in Table 3. The median PTX3 was significantly higher in patients with severe sepsis as compared to others (16.7 vs. 4.9 ng/ml, p<0.001) and in non-survivors (day 28 case fatality) as compared to survivors (14.1 vs. 5.1 ng/ml, p<0.001).

**Table 3:** Plasma pentraxin 3 (PTX3) values stratified by demographics and clinical data in patients admitted to the emergency room with suspected infection. N = 537.

	PTX3 (ng/ml) on admission. Stratification by clinical parameter				p-value
	Factor present		Factor absent		
Characteristic	N	median (quartiles)	N	median (quartiles)	
Age >60 years	312	6.3 (3.0–14.3)	225	4.0 (2.0–12.7)	**<0.001**
Gender (male)	310	5.2 (2.6–14.1)	227	5.8 (2.6–11.9)	0.887
Obesity (BMI^a^ ≥30)	119	4.4 (2.6–9.8)	271	6.5 (2.8–15.0)	**0.017**
Alcohol abuse^b^	25	12.7 (2.8–17.1)	512	5.3 (2.6–12.7)	0.088
Smoking (current smoker)	126	4.5 (2.5–15.5)	411	5.6 (2.6–13.0)	0.565
Diabetes (type 1 and 2)	81	5.8 (2.8–14.6)	456	5.3 (2.6–12.8)	0.134
Solid cancer	78	5.5 (2.9–11.4)	459	5.4 (2.6–14.1)	0.868
Malignancy	95	5.6 (2.9–11.6)	442	5.3 (2.6–14.1)	0.762
Rheumatic diseases	50	6.1 (2.8–11.4)	487	5.3 (2.6–14.0)	0.614
Chronic renal insufficiency^c^	18	7.0 (4.7–15.3)	519	5.3 (2.6–13.5)	0.336
Cardiovascular disease^d^	289	6.4 (2.9–14.3)	248	4.5 (2.3–12.1)	**0.002**
Continuous cortisone treatment^e^	59	7.6 (4.3–15.9)	478	5.0 (2.6–13.0)	**0.022**
**Clinical parameter (d 0–28)**					
Case fatality (d 28)	33	14.1 (5.2–53.6)	504	5.1 (2.6–12.5)	**<0.001**
Case fatality (d 90)	58	11.3 (4.5–39.7)	479	4.9 (2.6–12.2)	**<0.001**
Case fatality (1 year)	112	7.6 (3.8–30.8)	425	4.7 (2.5–12.2)	**<0.001**
ICU^f^ stay needed	42	11.6 (4.4 - 43.6)	495	5.2 (2.5–12.2)	**<0.001**
Hypotension^g^	28	17.8 (4.3–62.9)	509	5.3 (2.6–12.2)	**<0.001**
Vasopressors needed	19	14.2 (4.3–67.0)	518	5.3 (2.6–12.8)	**0.015**
DIC^h^	8	46.2 (21.8–153.9)	529	5.3 (2.6–12.8)	**0.001**
Decreased GCS^i^	26	15.4 (4.6–58.2)	511	5.3 (2.6–12.6)	**<0.001**
Needed mechanical ventilation	14	11.6 (2.8–53.9)	523	5.4 (2.6–13.0)	0.081
Needed C-PAP/bi-PAP^j^	22	9.0 (3.3–28.3)	515	5.3 (2.6–13.0)	0.064
Sepsis+organ dysfunction	49	16.7 (4.7–54.7)	488	4.9 (2.5–11.7)	**<0.001**
MOF^k^	10	46.2 (9.0–215.8)	527	5.3 (2.6–13.0)	**0.001**

a body mass index, data available on 390 patients.

b alcoholism was diagnosed or patient had previously been treated for alcohol-induced disease.

c plasma creatinine concentration constantly more than 170 µmol/l (5 patients had chronic dialysis treatment).

d continuous medication for cardiovascular disease (i.e. hypertension, arteriosclerosis or other cardiovascular disease).

e continuous systemic cortisone treatment (daily dose more than 10 mg of oral prednisolone).

f Intensive care unit.

g systolic blood pressure <90 mmHg or a reduction of 40 mmHg from baseline. No response to 500 ml intravenous fluid replacement.

h disseminated intravascular coagulation.

I Glasgow coma scale <15.

j continuous positive airway pressure/bilevel positive airway pressure.

k multi-organ failure.

**Figure 1:**
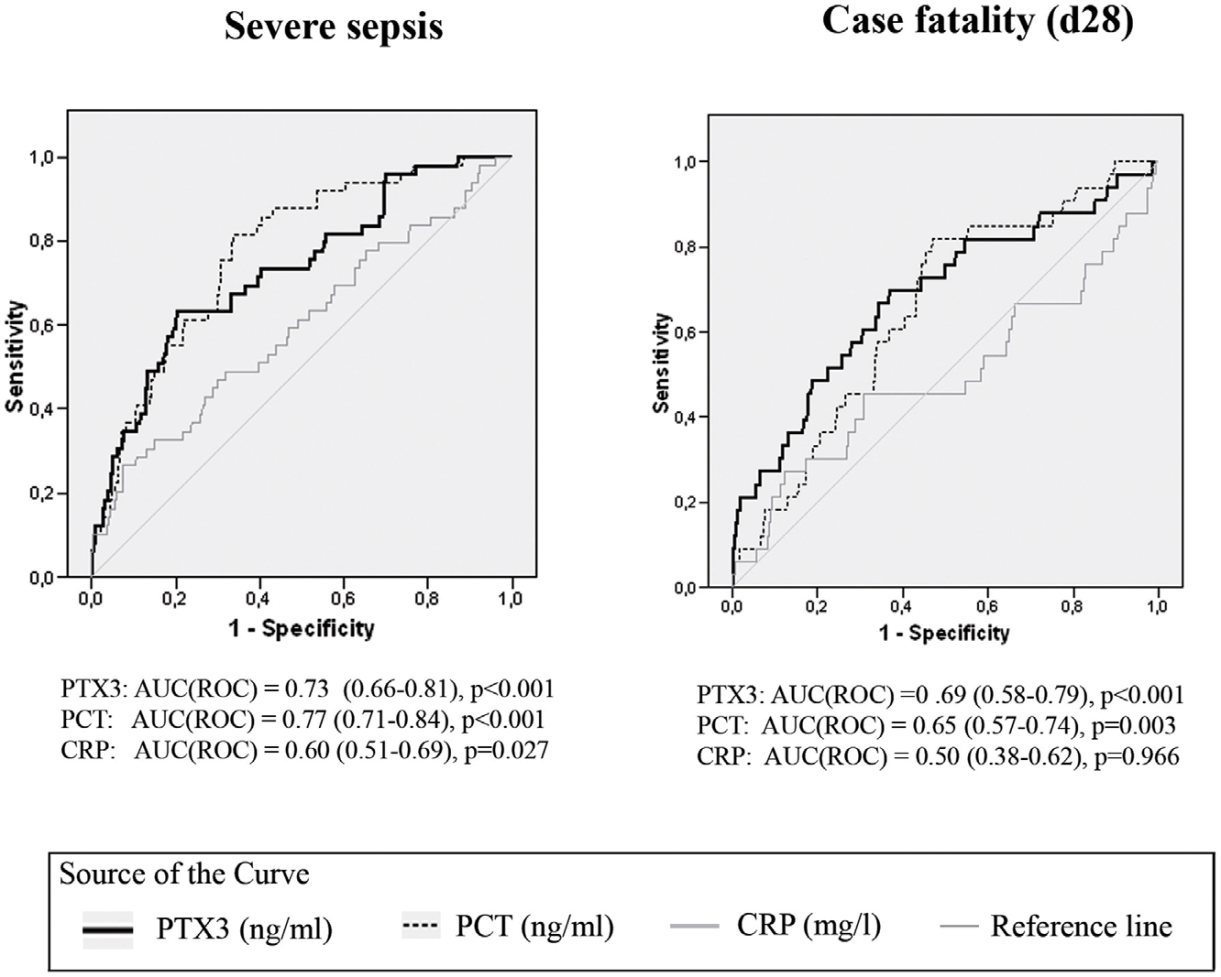
Receiver operating characteristic (ROC) curve for plasma levels of pentraxin 3 (PTX3), procalcitonin (PCT), interleukin-6 (IL-6) and C-reactive protein (CRP) detected on admission in relation to severe sepsis and case fatality (d28) in patients with suspected infection. AUC (ROC) (95% confidence interval, p<0.001.

The optimal cut-offs for PTX3, PCT and CRP in predicting severe sepsis between day 0 and day 28 and case fatality were estimated using ROC curves (Figure 1) and Youdeńs index. AUC^ROC^ for prediction of severe sepsis was 0.73 (95% CI 0.66–0.81, p<0.001) for PTX3. The AUC value for PCT was 0.77 (95% CI 0.71–0.84, p<0.001) and 0.60 for CRP (95% CI 0.51–0.69, p = 0.027). The optimal cut-off value for PTX3 in predicting severe sepsis was 14.1 ng/ml (specificity 80% and sensitivity 63%), for PCT 0.30 ng/ml (specificity 66% and sensitivity 82%) and for CRP 158 mg/l (specificity 70% and sensitivity 47%).

In predicting case fatality on day 28, AUC^ROC^ was 0.69 (95% CI 0.58–0.79, p<0.001) for PTX3, 0.65 (95% CI 0.57–0.74, p = 0.003) for PCT and 0.50 (95% CI 0.38–0.62, p = 0.966) for CRP. A PTX3 level of 7.7 ng/ml showed a sensitivity of 70% and specificity of 63% in predicting case fatality on day 28. For PCT the optimal cut-off level was 0.19 ng/ml (sensitivity 82% and specificity 63%). PTX3 predicted death also at 1 year follow up (table 3).

The clinical characteristics of patients stratified by PTX3 value as registered on admission are shown in Table 4. A high PTX3 level (≥14.1 ng/ml; optimal cut-off value for severe sepsis) was associated with day 28 case fatality and several endpoints reflecting the severity of the disease (need for ICU stay, hypotension and acute renal insufficiency (p<0.001)).

**Table 4:** Clinical characteristics of patients stratified by pentraxin3 (PTX3) value detected on admission.

*Clinical parameter*		*High PTX3, (≥14.1 ng/ml) (14.1 ng/ml), N = 130*	*Low PTX3 (<14.1 ng/ml)* *(<14.1 ng/ml), N = 407*	*OR (95% Cl)*	*p-value*
**Grouping variables (d 0–28)**	N	N (%)	N (%)		
Case fatality (d 28)	33	17 (13.1)	16 (3.9)	3.68 (1.80–7.51)	<0.001
Case fatality (d 90)	58	24 (18.5)	34 (8.4)	2.48 (1.41–4.37)	0.002
Case fatality (12 months)	112	37 (28.5)	75 (18.4)	1.76 (1.12–2.78)	0.015
Needed ICU^a^ stay	42	21 (16.2)	21 (5.2)	3.54 (1.87–6.729	<0.001
Hypotension^b^	28	17 (13.1)	11 (2.7)	5.42 (2.47–11.89)	<0.001
Needed vasopressors	19	10 (7.7)	9 (2.2)	3.69 (1.46–9.28)	0.006
Acute renal insufficiency^c^	16	13 (10.0)	3 (0.7)	15.0 (4.19–53.40)	<0.001
DIC^d^	8	7 (5.4)	1 (0.3)	28.08 (2.82–189.22)	0.004
Decreased GCS^e^	26	15 (11.5)	11 (2.7)	4.67 (2.10–10.5)	<0.001
Needed mechanical ventilation^f^	14	7 (5.4)	7 (1.7)	3.25 (1.12–9.45)	0.030
Needed C-PAP/bi-PAP	22	9 (6.9)	13 (3.2)	2.25 (0.94–5.40)	0.068
Severe sepsis	49	31 (23.9)	18 (4.4)	6.77 (3.64–12.59)	<0.001
MOF^g^	10	7 (5.4)	3 (0.7)	7.66 (1.95–30.05)	0.004
**Continuous variables on admission**		median (quartiles)	median (quartiles)		
Plasma C-reactive protein (mg/l)	537	147 (39–241)	101 (35–164)		0.001
Plasma procalcitonine (ng/ml)	537	1.19 (0.31–5.63)	0.11 (0.4–0.32)		<0.001
White cell count (10^9^/l)	523	12.7 (9.3–17.4)	10.0 (7.5–13.1)		<0.001
Platelet count (10^9^/l)	523	221 (164–281)	264 (210–346)		<0.001
Hemoglobin	523	129 (112–143)	129 (117–143)		0.567
Plasma creatinine (µmol/l)	487	93 (64–131)	79 (64–102)		0.11

Statistical differences between groups were tested by using Pearson Chi square (category) and Mann Whitney U test (continuous variables). Odds Ratio and 95% confidence limits with logistic regression analysis. The optimal cut-off level for PTX3 for severe sepsis was counted using ROC curve analysis and Youden’s index.

a intensive care unit.

b systolic blood pressure <90 mmHg or a reduction of 40 mmHg from baseline. No response to 500 ml intravenous fluid replacement.

c diuresis <30 ml/h at least 1 hour or continuous haemofiltration or acute dialysis treatment.

d disseminated intravascular coagulation.

e decreased Glasgow coma scale.

f needed mechanical ventilation otherwise than for surgery.

g Multi organ failure.

In a univariate model high PTX3, PCT and CRP values predicted severe sepsis when used as continuous or grouping variables. Alcohol abuse and continuous systemic cortisone treatment were also factors associated with severe sepsis in the univariate mode (data not shown). Parameters expressing significant associations were then combined in the multivariate model, first without and then with potential demographic confounders. Results are presented in Table 5.

**Table 5:** Multivariate logistic regression analysis evaluating the independent predictive value of pentraxin 3 (PTX3), procalcitonin (PCT) and C-reactive protein (CRP) for severe sepsis.

*Character*	*Odds ratio*	*95% Confidence limits*	*p*	
A. Parameters were included together in the logistic model without confounders (N = 537)				
PTX3≥14.1 ng/ml	3.36	1.70–6.67	<0.001	
PCT ≥0.30 ng/ml	5.24	2.25–12.22	<0.001	
CRP≥158 mg/l	0.94	0.49–1.82	0.851	
B. Parameters were taken for analysis together with statistically significant confounders (N = 537)				
PTX3≥14.1 ng/ml	3.02	1.50–6.01		0.002
PCT ≥0.30 ng/ml	5.55	2.37–13.00		<0.001
CRP≥158 mg/l	1.11	0.56–2.20		0.775
Alcohol abuse^a^	4.88	1.69–14.09		0.003
Continuous cortisone treatment^b^	4.20	1.82–9.70		<0.001

a alcoholism was diagnosed or patient had previously been treated for alcohol-induced disease.

b continuous systemic cortisone treatment (daily dose more than 10 mg of oral prednisolone.

The optimal cut-offs for these parameters were counted using ROC curve analysis and Youden's index.

High PTX3, PCT, age over 60 years, alcohol abuse, diabetes and continuous systemic cortisone treatment were associated with case fatality (d28) in the univariate model while the level of CRP was not (data not sown). These parameters were studied together in the multivariate model first without and then with potential demographic confounders. Results are shown in Table 6.

**Table 6:** Multivariate logistic regression analysis evaluating the independent predictive value of pentraxin 3 (PTX3) and procalcitonin (PCT) for 28-d case fatality.

*Character*	*Odds ratio*	*95% Confidence limits*	*p*
A. Parameters were included together in the logistic model without confounders (N = 537)			
PTX3≥7.7 ng/ml	2.55	1.13–5.74	0.024
PCT ≥0.19 ng/ml	3.54	1.36–9.18	0.009
B. Parameters were taken for analysis together with statistically significant confounders (N = 537)			
PTX3≥7.7 ng/ml	2.37	1.04–5.38	0.040
PCT ≥0.19 ng/ml	3.51	1.35–9.15	0.010
Age >60 years	3.02	1.11–8.19	0.030
Alcohol abuse^a^	6.01	1.60–22.67	0.008
Diabetes (type 1 and 2)	2.13	0.89–5.05	0.088
Continuous cortisone treatment^b^	2.28	0.93–5.59	0.073

a alcoholism was diagnosed or patient had been treated for alcohol-induced disease previously.

b continuous systemic cortisone treatment (daily dose more than 10 mg of oral prednisolone).

The optimal cut-offs for parameters were estimated using ROC curve analysis and Youden's index.

## Discussion

The results presented here show that high PTX3 levels on admission can be used to predict severe sepsis and case fatality in patients admitted to the emergency room with suspected infection. Both PTX3 and PCT remained independent predictors for severe sepsis and case fatality also after adjustment for potential confounders whereas high CRP did not.

In healthy persons the PTX3 concentration has been shown to be lower than 2 ng/ml, while PTX3 levels increase rapidly in response to inflammation and infection [19], [30]. A previous meningococcal study has shown that the levels of PTX3 peak already during the first hours after admission [24]. In a bacteremia study, PTX3 levels were high in the acute phase and normalized on recovery [23]. In another recent study conducted at the onset of febrile neutropenia in hematologic patients it was documented that PTX3 reached the maximal point earlier than CRP [31]. PTX3 may thus be used as an early biomarker in sepsis patients.

It is important to note that PTX3 is not a specific marker for bacterial infection. Elevated plasma PTX3 concentrations are seen in various inflammatory conditions. High PTX3 levels have been shown to correlate with unfavorable outcome in several conditions such as cardiovascular diseases [32], lung cancer [33] and polymyalgia rheumatica [34]. A high PTX3 concentration has also been shown to predict the severity of disease in dengue virus infection [35], leptospirosis [36] and epidemic nephropathy [37].

In our study PTX3 values were significantly higher in patients over 60 years and in those with obesity (body mass index (BMI) ≥30), cardiovascular diseases and continuous systemic cortisone treatment (daily dose over 10 mg oral prednisolone) compared to those without these risk factors. These findings are well in line with current knowledge of the inflammatory nature of these stages. Here was no difference in PTX3 levels in patients having solid cancer or hematological malignancies. The cohort included only 11 neutropenic hematologic patients.

In earlier studies, the utility of PTX3 as a sepsis marker has been compared with CRP. However, PTX3 has not previously been compared with PCT in the case of sepsis patients, although in many papers PCT has been shown to be a better prognostic marker than CRP [9], [10], [26]. In the present study, both PTX3 and high PCT seemed to be independent predictors of severe sepsis while CRP did not.

Several studies have shown the usefulness of PTX3 and CRP in assessing the severity of infection [21], [22], [23], [24], [38]. In one previous work high PTX3 has been shown to predict culture positive bloodstream infections and severe disease (the need for ICU treatment, longer hospital stay and acute congestive heart failure) in febrile patients admitted to emergency departments [20]. In critically ill ICU-patients the levels of PTX3 have correlated with the severity of disease and infection [21], [22]. High PTX3 has been found to be an early indicator of shock in severe meningococcal diseases [24]. The present findings confirm previous results on PTX3 in detecting severe disease, and show that PTX3 can be used in patients with suspected infection on admission. PTX3 may be used to predict many variables indicating severe sepsis, i.e. need for ICU stay, hypotension, acute renal insufficiency and need for mechanical ventilation, already in the emergency room setting. Thus, PTX3 may help in stratifying patients to target resources effectively.

High PTX3 and PCT levels were shown here to be independent predictors of case fatality on day 28 when plasma samples were taken on admission together with blood cultures from patients with suspected infection. In one earlier study the maximum PTX3 value on days 1–4 after bacteremia diagnosis was shown to be a predictor of case fatality (d28) [23]. Another recent study has shown that high levels of plasma PTX3 persisting over the first five days after onset of severe sepsis and septic shock are associated with mortality, but in contrast to the present findings, not the PTX3 value on day 1 [22].

In our study only a weak negative correlation between PTX3 levels and platelet count was documented. Previously it has been shown that PTX3 can up-regulate tissue factor in activated monocytes - a link between inflammation and clotting activity [39]. This has also been shown in previous sepsis studies [22], [24], and PTX3 may be involved in the pathological coagulation process in these conditions. High PTX3 level may also reflect the role of pentraxins in the clearance of apoptotic cells [40]. Early elevated PTX3 levels are associated with more severe forms of sepsis, number of organ failures and poor outcome [22], [41].

Some limitations must be conceded here. There were some confounding factors in our study. Our study was designed to test indicators of severe sepsis and case fatality in an emergency room setting in an unselected patient population with suspected infection. A clinician had made the decision to take blood cultures from all patients in the emergency room and plasma samples were taken simultaneously. We carefully sought to control for confounding factors and would consider our results to reflect the real life situation in an emergency room setting [26]. We could not control the individual time delay of patients from the onset of symptoms to hospital admission. Only one sample was studied. The study was not designed to study the effects of antimicrobial therapy on PTX3 levels. Study sample was taken before antibiotic therapy in hospital but about quarter of patients had some antimicrobial treatment before admission to hospital.

In our protocol patients were enrolled only from Sundays to Wednesdays, but comparing this study population to a prior evaluation made in 1551 consecutive patients before the study commenced, no difference was seen in age, gender and the rate of positive blood cultures or case fatality rate on day 28 between the study and the target population. Also in the severe sepsis group the mortality rate and distribution of infection foci were in concord with the findings in a Finnish multicentre sepsis study [42].

The optimal PTX3 cut-off point for severe sepsis here was about the same as one would expect from earlier studies but the optimal cut-off for day 28 case fatality was surprisingly low, giving too low specificity for clinical work. The results here show that PTX3 possesses statistically significant capacity as a prognostic marker also in the emergency room setting but for better specificity the cut-off level should be higher.

### Conclusions

We showed here that high levels of PTX3 in plasma as well as high levels of PCT can be used as prognostic markers in patients with suspected infection admitted to the emergency room. High PTX3 and PCT were independent predictors for severe sepsis between day 0 and 28 and case fatality on day 28 after admission.
